# dbPTM 2016: 10-year anniversary of a resource for post-translational modification of proteins

**DOI:** 10.1093/nar/gkv1240

**Published:** 2015-11-17

**Authors:** Kai-Yao Huang, Min-Gang Su, Hui-Ju Kao, Yun-Chung Hsieh, Jhih-Hua Jhong, Kuang-Hao Cheng, Hsien-Da Huang, Tzong-Yi Lee

**Affiliations:** 1 Department of Computer Science and Engineering, Yuan Ze University, Taoyuan 320, Taiwan; 2 Department of Biological Science and Technology, National Chiao Tung University, Hsinchu 300, Taiwan; 3 Institute of Bioinformatics and Systems Biology, National Chiao Tung University, Hsinchu 300, Taiwan; 4 Innovation Center for Big Data and Digital Convergence, Yuan Ze University, Taoyuan 320, Taiwan

## Abstract

Owing to the importance of the post-translational modifications (PTMs) of proteins in regulating biological processes, the dbPTM (http://dbPTM.mbc.nctu.edu.tw/) was developed as a comprehensive database of experimentally verified PTMs from several databases with annotations of potential PTMs for all UniProtKB protein entries. For this 10th anniversary of dbPTM, the updated resource provides not only a comprehensive dataset of experimentally verified PTMs, supported by the literature, but also an integrative interface for accessing all available databases and tools that are associated with PTM analysis. As well as collecting experimental PTM data from 14 public databases, this update manually curates over 12 000 modified peptides, including the emerging *S*-nitrosylation, *S*-glutathionylation and succinylation, from approximately 500 research articles, which were retrieved by text mining. As the number of available PTM prediction methods increases, this work compiles a non-homologous benchmark dataset to evaluate the predictive power of online PTM prediction tools. An increasing interest in the structural investigation of PTM substrate sites motivated the mapping of all experimental PTM peptides to protein entries of Protein Data Bank (PDB) based on database identifier and sequence identity, which enables users to examine spatially neighboring amino acids, solvent-accessible surface area and side-chain orientations for PTM substrate sites on tertiary structures. Since drug binding in PDB is annotated, this update identified over 1100 PTM sites that are associated with drug binding. The update also integrates metabolic pathways and protein–protein interactions to support the PTM network analysis for a group of proteins. Finally, the web interface is redesigned and enhanced to facilitate access to this resource.

## INTRODUCTION

Post-translational modification (PTM), which involves the attachment of chemical groups, such as phosphate, acetyl, methyl or oligosaccharides, to the amino acid side chains of proteins, is important in signal transduction and apoptosis (as in phosphorylation), transcriptional regulation (by acetylation and methylation) and cell–cell and cell–matrix interactions (such as glycosylation) (1,2). Other types of PTM involve covalent linkage to ubiquitin or a ubiquitin-like protein, as in ubiquitylation and SUMOylation (3). The formation of disulfide bonds from cysteine residues may also be referred to as a post-translational modification (4). Contemporary research has implicated the dysregulation of PTMs in severe pathological events, including cancer, disease and drug resistance, motivating a thorough investigation of protein modification dynamics (5–10). Mass spectrometry (MS)-based experiments provide a practical means of the site-specific identification of PTMs in proteomics (11). High-throughput MS or MS/MS-based proteomics has motivated an increasing number of studies of large-scale modified proteomes (1). Thus, many databases of modified peptides for specific PTM types, including O-GLYCBASE (12), dbOGAP (13), PhosphoSitePlus (14), Phospho.ELM (15), PhosPhAt (16), UbiProt (17) and PupDB (18), have been developed. A growing number of proteomic studies have reported that the emerging oxidative modifications, a major class of PTMs that involve reactions between amino acid residues and reactive oxygen species or reactive nitrogen species (19), have crucial roles in the regulation of redox-related pathways (20). With this, two public databases, dbSNO (21,22) and dbGSH (23), were designed by manually curating *S*-nitrosylated and *S*-glutathionylated peptides, respectively, from research articles.

Owing to the importance of PTMs in regulating cellular processes, NetworKIN (24) and RegPhos (25,26) have utilized phosphoproteome data to gain insight into kinase-mediated signaling networks. In addition, given the biological significance of E3 ligases in ubiquitin-mediated protein degradation (27), E3Net (28) is a collection of 1671 E3-substrate relations between 493 E3s and 1277 substrates in 42 organisms. Sakiyama *et al*. built a database of proteins that are involved in the ubiquitin signaling cascade across species (29). More than 200 different types of PTM have been identified by MS-based proteomics so several resources (30–33) have been developed to accumulate these multiple PTM types with functional annotations. Owing to the difficulty of collecting heterogeneous data from various PTM resources, dbPTM (34) was developed by systematically integrating experimentally verified PTMs from various resources and comprehensively annotating the putative PTM substrate sites for all UniProtKB (35) protein entries. Since an increasing number of site-specific PTMs are being obtained through high-throughput MS/MS-based proteomics, version 3.0 of dbPTM was extended as an informative resource for investigating the substrate site specificity and functional association of PTMs (36).

In its 10th anniversary, dbPTM is updated as an integrated resource for PTMs, providing not only a comprehensive dataset of experimentally verified PTMs that are supported by the literature but also an integrative platform for accessing all available databases and tools that are associated with PTM analysis. In addition to collecting experimental PTM data from public databases, this update manually curates more than 12 000 PTM peptides, including the emerging *S*-nitrosylation, *S*-glutathionylation and succinylation, from approximately 500 research articles which were extracted by text mining. This update develops an integrative platform for PTM analyses by integrating all available databases and tools that are associated with over 20 PTM types. Given the availability of numerous PTM prediction methods, this update further compiles a non-homologous benchmark dataset to evaluate the predictive power of PTM prediction tools in an attempt to provide suggestions to users who need to predict PTM sites with high sensitivity (Sn), high specificity (Sp) or balanced Sn and Sp. In this update, all manually curated PTM peptides are mapped to protein entries of the Protein Data Bank (PDB) (37) based on UniProtKB ID and sequence identity, which enables dbPTM to provide information about spatial amino acid composition, solvent-accessible surface area, structurally neighboring amino acids and the orientation of side chains at PTM substrate sites on protein tertiary structures. In particular, the side-chain orientations of the amino acids that structurally surround the PTM substrate sites were determined to elucidate the functional roles and binding effects of the amino acids that neighbor the substrate sites. Moreover, this update allows users to submit a group of proteins to construct a full map of regulatory network for a specific PTM type. The updated dbPTM is now accessible at http://dbPTM.mbc.nctu.edu.tw/.

## IMPROVEMENTS

**Figure 1.**
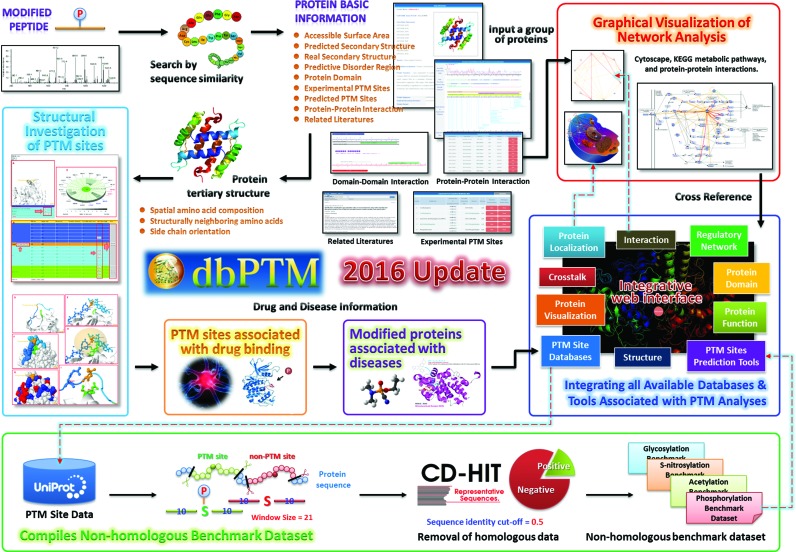
The highlighted improvements and advances in dbPTM update 2016.

Figure 1 presents selected improvements and advances that are provided by the dbPTM update 2016, including (i) an update of the data on site-specific PTMs, (ii) the establishment of an integrative platform and benchmark dataset for PTM analysis, (iii) the development of an interactive viewer for the structural characterization of PTM substrate sites, (iv) data integration to elucidate diseases and drugs that are associated with PTM substrate sites and (v) the construction of PTM regulatory networks using metabolic pathways and protein–protein interactions. To facilitate a study of PTMs and their functions, the web interface has been redesigned and enhanced. This resource also provides information on the literature related to PTMs, protein domains, functional associations and the substrate motifs of PTM sites. Details of each improvement follow.

### Data update concerning site-specific PTMs

Supplementary Figure S1 presents the flowchart for data enhancement in dbPTM 2016. The large-scale site-specific identification of PTM peptides by MS/MS-based proteomics has motivated the development of databases that are dedicated to the accumulation of experimentally verified data concerning a specific PTM or multiple PTMs. Owing to the difficulty of collecting heterogeneous data from a variety of PTM databases, dbPTM has been developed as a systematic pipeline for automatically extracting experimentally verified PTMs from all available PTM-related resources. Supplementary Table S1 summarizes 14 integrated PTM databases. This update manually curates more than 12 000 modified peptides, including the emerging *S*-nitrosylated, *S*-glutathionylated and succinylated peptides, from about 500 research articles, which were retrieved by text mining. Since various proteomic identification experiments have been conducted, a text-mining method was developed to retrieve research articles that potentially describe the site-specific identification of modified peptides. Firstly, the PTM-related research or review articles were systematically retrieved by querying PTM-related keywords against the fields ‘Title’ and ‘Abstract’ in the PubMed literature database. Then, the full-length articles were manually reviewed to extract modified peptides along with the corresponding substrate residues. To determine the precise locations of PTM substrate sites within a full-length protein sequence, all of the collected PTM peptides are mapped to UniProtKB protein entries based on database identifier (ID) and sequence identity. Finally, each mapped PTM site is associated with at least one article (PubMed ID). Modified peptides that could not be mapped to a protein sequence in UniProtKB were removed from the dbPTM database.

### Establishment of integrative platform and benchmark dataset for PTM analysis

Owing to the biological significance of PTMs in regulating cellular processes, an increasing number of resources have been developed for PTM analysis, including the data warehousing of PTM sites, the computational prediction of PTM sites, the structural investigation of PTM substrate sites and the reconstruction of PTM regulatory networks. However, given a protein sequence of interest, users commonly have difficulty in making a full study of PTMs by surveying suitable PTM-related databases or tools on the internet. Therefore, this update includes the design of an integrative web interface that enables users to access all online databases and tools that are associated with approximately 20 types of PTM, such as phosphorylation, glycosylation, acetylation, methylation, ubiquitylation, sumoylation, palmitoylation and *S*-nitrosylation. Supplementary Table S2 lists the number of integrated databases, database names, number of integrated tools and tool names for each PTM type.

Since MS/MS-based experiments are labor-intensive, a range of computational methods (38–51) have been developed to identify putative PTM sites based on protein sequences. Since numerous PTM prediction methods are available, determining the best prediction tool based on only cross-validation performance is difficult. Although most related studies have provided independent results of tests of prediction methods, no standard dataset exists for the evaluation of the predictive power of various PTM prediction tools. Therefore, this update provides a non-homologous benchmark dataset to evaluate the predictive power of PTM sites prediction tools and thereby helps users to predict PTM sites with high Sn, high Sp or balanced Sn and Sp. Firstly, a window length of 2*n* + 1 was used to extract sequence fragments that were centered at the experimentally verified PTM sites and contained *n* upstream and *n* downstream flanking amino acids. For a modified protein, the sequence fragments that contain a window length of 2*n* + 1 (*n* = 10) amino acids and are centered at a specified modified residue (such as an ubiquitylated lysine residue) were regarded as the positive dataset. The sequence fragments that contain a window length of 2*n* + 1 amino acids and are centered at a non-modified residue of the same type (such as a non-ubiquitylated lysine residue) were regarded as the negative dataset. Then, the CD-HIT program (52) was employed to remove homologous sequence fragments from the positive and negative datasets. CD-HIT is an effective tool for clustering protein sequences based on a specified sequence similarity value. One sequence was chosen herein to represent each cluster. Based on the analysis of sequence fragments, some negative data may have been identical to positive data, potentially leading to false-positive or false-negative predictions. Therefore, CD-HIT was applied a second time, by running cd-hit-2d across positive and negative training data with 100% sequence identity. Supplementary Table S3 presents statistics about the benchmark datasets for several PTM types after the homologous fragments were eliminated using CD-HIT, based on a 50% sequence identity.

### Development of interactive viewer for structural characterization of PTM substrate sites

With the steadily growing number of PTM sites that have been experimentally confirmed using high-throughput MS-based proteomic techniques, interest in the structural environment of PTM substrate sites (48,53), including spatial amino acid composition, solvent-accessible surface area, structurally neighboring amino acids and the orientation of side chains around PTM substrate sites, has been increasing. In this update, X-ray crystal protein structures with experimental resolution of better than 2.5 Å were utilized to elucidate the spatial context of PTM substrate sites on protein tertiary structures. Since only a few protein structures involve the covalent attachment of chemical groups to the side chain of target residues, all of the experimentally verified PTM peptides are mapped to the protein entries of the PDB to determine the exact PTM substrate sites on tertiary structures, based on UniProtKB cross-references and sequence identity (with 100% similarity). As presented in Supplementary Table S4, a total of 25 835 PTM sites were thus mapped to the protein three-dimensional (3D) structures of PDB. Dictionary of protein secondary structure (DSSP) (54) was then adopted to calculate the solvent-accessible surface area and to standardize the secondary structure of PDB entries with the mapped PTM substrate sites. Sometimes, identifying the substrate motif from linear sequences is difficult (44); therefore, this update uses a radial cumulative propensity plot (55) to represent the spatial amino acid composition of a specific PTM site, revealing the abundance of 20 amino acids in the spatial vicinity of PTM substrate sites. A spatial amino acid composition was determined for all mapped PTM sites by calculating the relative frequencies of the 20 amino acids within radial distances from 2 to 10 Å of the modified residues.

With respect to the structural characterization of PTM substrate sites, sequentially and spatially neighboring amino acids are displayed with different colors on PDB 3D structures using JSmol software (56). The side chain orientations of the amino acids that spatially surround the PTM substrate sites are determined to examine the functional roles and drug binding effects of the spatially neighboring amino acids to the substrate sites of PTMs (57). With respect to an N-linked glycosylation substrate site *p* and its spatially neighboring amino acid *k*, the vector *S_k_* from the Cα atom to the nitrogen of N-linked glycosylated asparagine (*p*) is defined as:

(1) \begin{equation*} {S}_{k} = {X}_{p}^{{\rm SG}} - {X}_{k}^{{\rm C}\alpha } \end{equation*}

where ${X}_{p}^{{\rm SG}}$ and ${X}_{k}^{{\rm C}\alpha }$ denote the crystallographic positions of the nitrogen in glycosylated asparagine *p* and the Cα atom in residue *k*, respectively. As displayed in Supplementary Figure S2, the direction of the side chain of a spatially neighboring amino acid *k* is given by the vector *V_k_* from its Cα atom to the functional atom (58):

(2) \begin{equation*} {V}_{k} = {X}_{k}^{\rm F} - {X}_{k}^{{\rm C}\alpha } \end{equation*}

where ${X}_{k}^{\rm F}$ and ${\rm X}_{\rm k}^{{\rm C}\alpha }$ are the crystallographic positions of the functional atom and the Cα atom, respectively, in residue *k*. The angle $\theta _k$ between vectors *S_k_* and *V_k_*, which specifies the effect of the side chain of a spatially neighboring amino acid *k* on the substrate asparagine residue, is computed as,

(3) \begin{equation*} \theta _k = \arccos \frac{{{ S}_{ k} \cdot V_{ k} }}{{\left\| { S_{ k} } \right\|\left\| { V_{ k} } \right\|}} \end{equation*}

For a spatially neighboring amino acid *k*, if the angle $\theta _k$ is less than 80°, then the amino acid *k* is defined as a functional residue to the asparagine residue on the N-linked glycosylation (58). To facilitate the structural investigation of protein modification sites, all of the structural characteristics were graphically represented in the JSmol program.

### Integration of data on diseases and drugs associated with PTM substrate sites

Many proteins undergo PTMs that involve physical or chemical changes to their side chains, causing cancer or other diseases; other PTMs may be used diagnostically (5–10). Accordingly, the disease annotations in the KEGG Disease Database (59), the Online Mendelian Inheritance in Man database (OMIM) (60) and Human Protein Reference Database (HPRD) (61) were integrated to identify associations between diseases and PTM-associated proteins. Despite the fact that more than 60% of eukaryotic proteins undergo PTMs during or after protein biosynthesis, little is known about the frequency and local effects of PTMs close to drug or inhibitor-binding sites. A phosphorylation site within 12 Å of a small molecule-binding site is reportedly likely to alter the binding affinity of this small molecule (62). Therefore, the drug annotations in DrugBank (63) were combined with all available PDB entries that contained keywords ‘drug,’ ‘inhibitor,’ ‘agonist’ or ‘antagonist.’ After all experimentally verified PTM sites were mapped to PDB structures, the PTM sites whose side chains are located within 10 Å of a drug-binding site were regarded as drug binding-associated PTMs. Based on a large-scale screening of PTM sites and drug-binding sites in PDB, over 1100 PTM sites that are associated with drug-binding sites were identified. Additionally, if a modified protein was found to contain the 3D structures with PTM sites and without PTM sites, a molecular docking tool could be utilized to calculate the binding effect of a drug to a specific PTM site based on a protein tertiary structure.

### Construction of PTM regulatory networks using metabolic pathways and protein–protein interactions

Many studies (24–26,28–29) have suggested that protein modification is critical to the regulation of cellular signaling and metabolic pathways. Hence, one of the goals of this update is to present a full investigation of PTM regulatory networks for a group of genes/proteins of interest. This update integrates information about metabolic pathways and protein–protein interactions (PPIs) to perform a network analysis of a specific type of PTM. The information about metabolic pathway is taken from the pathway maps in KEGG. The information on experimentally verified physical interactions is taken from more than ten PPI databases (listed in Supplementary Table S5) and integrated into dbPTM. With respect to the example of *S*-nitrosylation, presented in Supplementary Figure S3, the dbPTM was sought to identify *S*-nitrosylated annotations for a group of proteins of interest and the proteins were then mapped onto metabolic pathways using the Cytoscape program (64). The PPIs that are associated with the proteins of interest were utilized to discover new members that have the potential of being involved in a mapped metabolic pathway. To make the construction of PTM regulatory networks feasible, a graph theory (25) was applied to formalize the networks based on a KEGG pathway map. In particular, the catalytic kinases were annotated by the network viewer to study the protein phosphorylation networks (Supplementary Figure S4).

## DATA CONTENT AND UTILITY

### Statistics about PTM sites in dbPTM 2016

In an attempt to provide the most comprehensive data on PTM sites, this update not only accumulates experimentally verified PTMs from 14 external PTM-related databases but also includes manually curated MS/MS-identified PTM peptides from approximately 500 research articles. After the redundant data from these heterogeneous resources were eliminated, a total of 610 037 experimentally verified PTM sites were stored in dbPTM using a structured database management system. The use of high-throughput MS/MS-based proteomics in the site-specific identification of modified peptides has motivated the obtaining of a rapidly rising number of experimental data concerning several types of PTM, including ubiquitylation, N-linked glycosylation, acetylation, palmitoylation, *S*-nitrosylation, *S*-glutathionylation and the emerging succinylation. Table 1 provides the number of obtained experimental data concerning each PTM type. Protein phosphorylation is the most popular research object and is associated with the most abundant data on experimentally verified substrate sites (258 654 sites). The dbPTM includes not only the experimental PTM sites, but also a total of 546 911 putative PTM sites that were taken from UniProtKB. Additionally, based on the investigation of disease associations with various PTMs, the distribution of the top ten diseases among six representative PTM types is provided. As presented in Supplementary Table S6, a total of 1690 phosphorylated proteins are associated with diseases, including mental retardation (66 proteins), cardiomyopathy (42 proteins), immunodeficiency (34 proteins), Charcot–Marie–Tooth disease (29 proteins), spinocerebellar ataxia (28 proteins), deafness (20 proteins), spastic paraplegia (20 proteins), diabetes mellitus (19 proteins), amyotrophic lateral sclerosis (18 proteins), and retinitis pigmentosa (18 proteins).

**Table 1.** Data statistics of experimental and putative PTM sites in dbPTM 2016.

PTM type	Number of experimental substrate sites	Number of experimental substrate sites from UniProtKB	Number of putative substrate sites from UniProtKB
Phosphorylation	258 654	41 083	96 915
Ubiquitylation	111 207	-	-
N-linked glycosylation	103 016	5172	100 846
Acetylation	35 527	8829	53 022
O-linked glycosylation	5729	1150	3204
Amidation	4449	1886	1309
Hydroxylation	3436	1504	5767
Methylation	8096	1263	23 070
Pyrrolidone carboxylic acid	1679	629	748
SUMOylation	1638	-	-
Gamma-carboxyglutamic acid	1262	-	-
4-carboxyglutamate	399	399	868
Palmitoylation	5576	-	-
Sulfation	1019	-	-
Sulfotyrosine	186	186	839
Myristoylation	1454	-	-
C-linked glycosylation	255	152	59
Prenylation	1459	-	-
Nitration	190	51	280
Deamidation	231	64	380
S-nitrosylation	4165	64	459
Oxidation	1126	-	-
ADP-ribosylation	314	17	1082
N6-succinyllysine	4637	1381	5571
Formylation	190	64	40
GPI anchoring	0	-	-
N6-lipoyllysine	19	19	6357
Methyl ester	87	87	914
N6-crotonyllysine	342	342	213
Methionine sulfoxide	52	38	305
N6-glutaryllysine	43	43	81
4-aspartylphosphate	29	29	8732
Pyridoxal phosphate	6371	23	148 475
Bromination	90	30	57
N6-malonyllysine	200	33	167
Citrullination	220	113	319
N6-carboxylysine	1608	37	20 848
Glutathionylation	4119	31	35
FAD	183	1	766
Pupylation	268	-	-
Others	40 512	370	65 183
**Total**	**610 037**	**65 090**	**546 911**

### An integrative platform for PTM analysis

**Figure 2.**
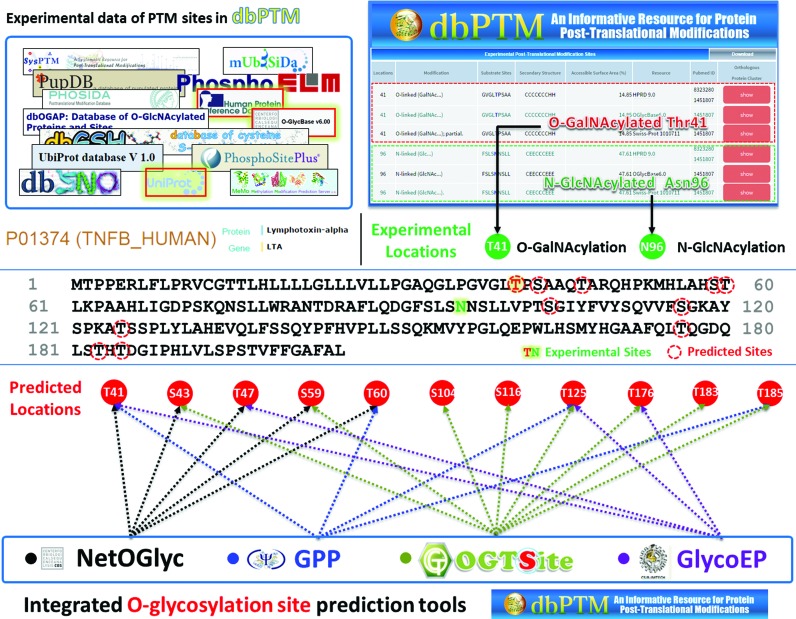
A case study of integrative protein glycosylation analyses for lymphotoxin-alpha (LTA).

In this update, the web interface is enhanced to enable users to browse and search efficiently for their proteins of interest. Supplementary Figure S5 presents the data content of a typical dbPTM query, including basic information, a graphical visualization of PTM sites with structural characteristics and functional domains, a table of experimental PTM sites with relevant literature, information on the orthologous conservation of PTM substrate sites, PPIs and domain–domain interactions, and references to literature on PTMs. To provide an integrated resource for PTM analysis, as displayed in Supplementary Figure S6, this update provides an integrative platform for accessing all online resources that are associated with PTM analysis, including PTM databases, PTM site prediction tools, 3D structure viewers and network investigators. Supplementary Table S2 provides a total of 71 databases and 116 tools that are associated with over 20 PTM types. Given the protein sequence of lymphotoxin-alpha, dbPTM efficiently provides comprehensive annotations of experimental PTM sites, including O-GalNAcylated Thr41 and N-GlcNAcylated Asn96, with references to supporting literature (65). The integrated glycosylation site prediction tools can be adopted to identify the putative substrate sites of protein glycosylation. In Figure 2, a total of 11 potential glycosylation sites, including the experimental O-GalNAcylated Thr41, are predicted by four eukaryotic glycosylation prediction tools—NetOGlyc (66), GPP (67), GlycoEP (68) and OGTSite (40). Eight of the 11 putative sites are detected by at least two prediction tools, which support a preliminary analysis for the further verification of protein glycosylation.

### Enhanced web interface for structural investigation of PTM substrate sites

**Figure 3.**
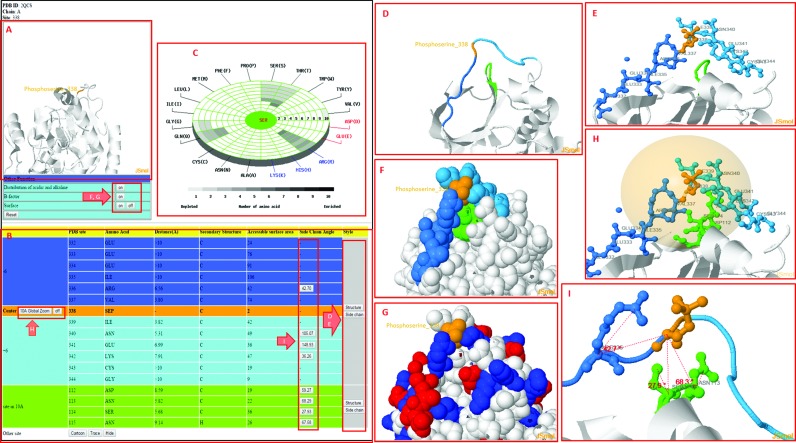
A case study of exploring the spatial context of the phosphorylation substrate site of serine (Ser338) on the protein 3D structure (PDB ID: 2QCS) of cAMP-dependent protein kinase catalytic subunit alpha (UniProtKB ID: KAPCA_MOUSE).

This update includes a newly designed interactive platform with which users can access the structural contexts of PTM substrate sites based on protein tertiary structures of PDB. Figure 3 presents a case study of the phosphorylation substrate site of serine (Ser338) on the protein 3D structure (PDB ID: 2QCS) of cAMP-dependent protein kinase catalytic subunit alpha (UniProtKB ID: KAPCA_MOUSE). Figure 3A shows an overview of the phosphorylation substrate site (Ser338) on the protein 3D structure. Figure 3B presents a table of sequentially and structurally neighboring amino acids, including information on the orientations of the side chains. Figure 3C provides a radial cumulative propensity plot of the spatial amino acid composition of the phosphorylation substrate site (Ser338). Arginine (Arg) is the most abundant amino acid in the spatial vicinity of the phosphorylation substrate site (Ser338). Figure 3D displays the sequentially and structurally neighboring amino acids on the 3D structure. The sequentially upstream (from positions -6 to -1) and downstream (from +1 to +6) amino acids are colored in blue and light blue, respectively. The structurally neighboring amino acids, whose radial distance to the side chain of Ser338 is less than 10 Å, are shown in green on the 3D structure. Figure 3E presents the side chains of the sequentially and structurally neighboring amino acids on the 3D structure. Figure 3F shows the surface area of Ser338, as well as the sequentially and structurally neighboring amino acids, to support an analysis of solvent accessibility. In Figure 3G, the acidic residues (K, R and H) and basic residues (D and E) are marked in blue and red, respectively, to elucidate the structural acid-based motif (69) that surrounds the PTM substrate site. Figure 3H shows the spatial vicinity within 10Å of the C-alpha atom of Ser338. Figure 3I presents the top three nearest amino acids (Asn113, Ser114 and Arg336) and information on the orientation of the side chains to support the investigation of the structurally neighboring amino acids. For instance, the Ser114 residue, which is close to the phosphorylation site (Ser338), contains a side chain with an angle of 27.9°. Ser114 residue may thus significantly influence the binding of phosphate to Ser338.

### Case study of PTM sites associated with drug binding

**Figure 4.**
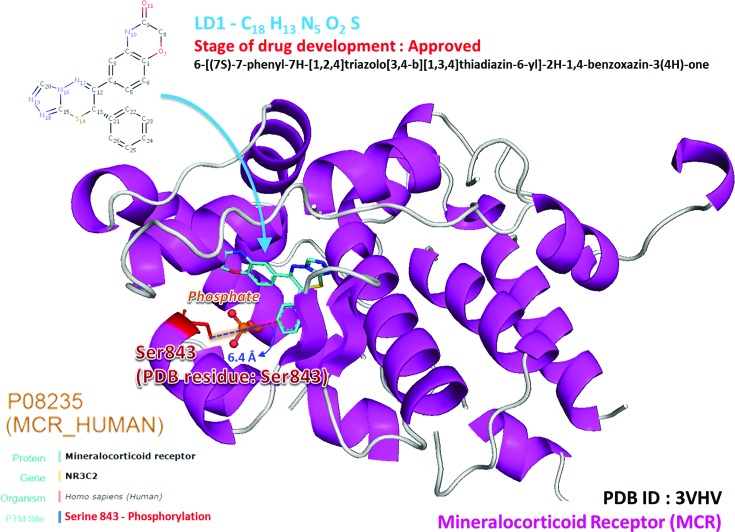
A case study of phosphorylation site (Ser843) associated with drug binding on mineralocorticoid receptor (MCR).

**Figure 5.**
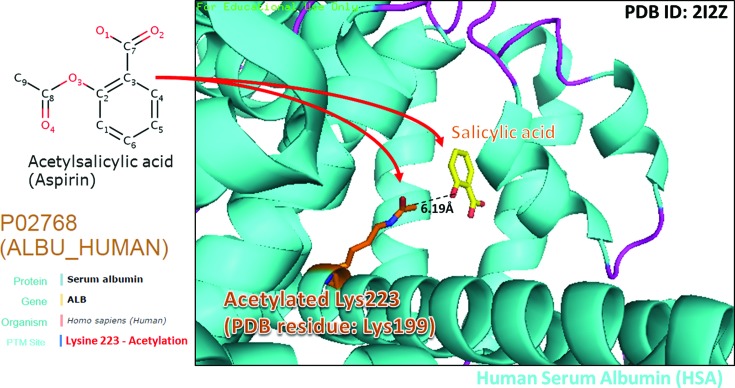
A case study of acetylation site (Lys199) associated with drug binding on human serum albumin (HSA).

Based on a large-scale screening of PTM substrate sites and drug-binding sites in PDB, dbPTM includes over 1100 PTM substrate sites that are associated with drug binding. Supplementary Table S7 presents the number of PTM sites that are associated with drug binding for each PTM type. Protein phosphorylation is the PTM with the most data concerning the association of substrate sites with drug binding, and it is followed in this regard by protein ubiquitylation. Figure 4 presents a case study of a phosphorylation site (Ser843) that is associated with drug binding on the mineralocorticoid receptor (MCR). Since the side chain of Ser843 is located close to (6.4 Å) the binding site of both the agonist and the inhibitor of the MCR, according to the data in dbPTM the phosphorylation of Ser843 influences the binding affinity of drugs. The phosphorylation of MCR at Ser843 reportedly reduces binding affinity for the natural agonist and inactivates itself (70). Figure 5 provides a case study of an acetylation site (Lys199) that is associated with drug binding on human serum albumin (HSA). HSA is the most abundant plasma protein in the human body and is critically involved in drug transport and metabolism (71). According to the annotation from OMIM, HSA is related to hyperthyroxinemia (OMIM ID: 615999) and analbuminemia (OMIM ID: 616000). According to the data in dbPTM, acetyllysine (Lys199) is located near (6.19 Å) the binding site of salicylic acid (DrugBank ID: DB00936). Aspirin (DrugBank ID: DB00945) reportedly transfers an acetyl group to Lys199 and is hydrolyzed into salicylic acid by HSA (71). This structural investigation not only reveals the conformational plasticity of HSA in drug binding but also the modulation of HSA drug interaction.

### Case study of exploring protein O-glycosylation network for a group of proteins

**Figure 6.**
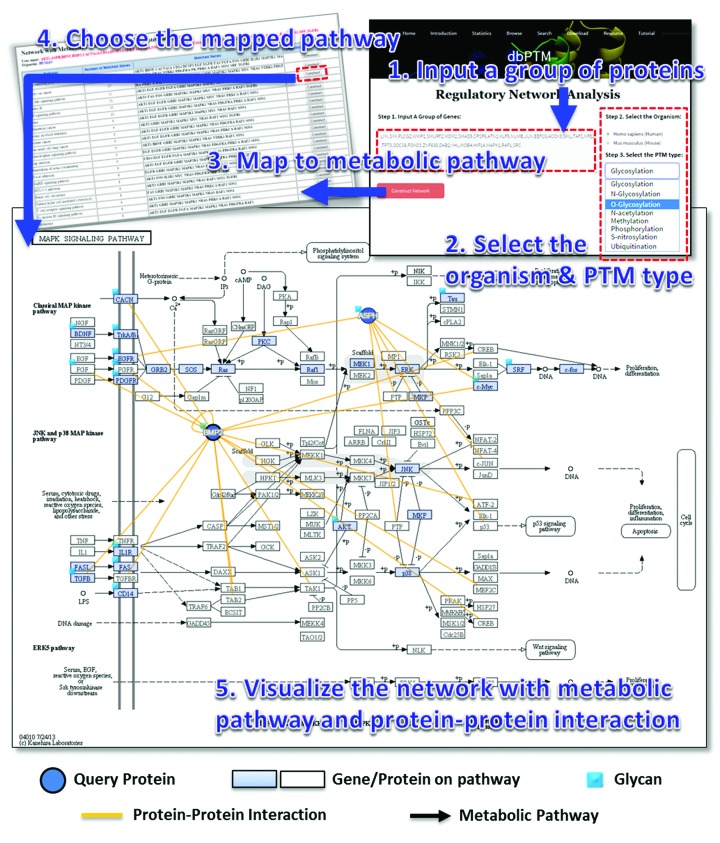
A case study of exploring protein O-glycosylation networks for a group of 20 proteins.

This update includes a newly designed interactive interface for discovering a regulatory network of modified proteins based on information about both metabolic pathways and PPIs. Figure 6 presents a case study of protein O-glycosylation networks for a group of 20 proteins. In network visualization, the query proteins that can be mapped to a member of a metabolic pathway are represented as light blue squares. The query proteins that have O-glycosylation sites are shown with a small light blue square. In this case, most of query proteins have O-glycosylation sites and can be mapped to the Mitogen-activated protein kinases (MAPK) signaling pathway. The query proteins that could not be mapped to a specific member of a metabolic pathway are represented by blue circles; they include BMP2, ASPH, GLA, ACE2 and AFM in this case. The PPIs that are associated with the query proteins are displayed as yellow lines. Given that the query proteins interact with the members of a well-known signaling pathway, their upstream and downstream targets can be used to find new members that have the potential to be involved in the mapped pathway (22). Taken together, the O-glycosylated BMP2 and ASPH, which undergo many interactions with pathway members, may be involved in the MAPK signaling pathway by participating in an interplay between protein glycosylation and phosphorylation. This network investigation may support a preliminary analysis based on which the regulatory network of a specific protein modification can be mapped.

## DISCUSSIONS AND CONCLUSION

The present expansion of the dbPTM database enhances its usefulness for researchers into the impact of PTMs on protein function, disease association, drug binding and cellular processes. The improved web interface enables both wet-lab biologists and bioinformatics researchers efficiently to increase their knowledge of protein post-translational modifications. With the goal of developing an integrated resource for PTM analysis, a total of 71 databases and 116 tools that are associated with over 20 types of PTM were gathered to provide an integrative interface for users. However, the increasing number of PTM prediction tools raises a difficulty in comparing their predictive power based on different training datasets. Therefore, this update compiles a sufficiently large non-homologous benchmark dataset for nine types of PTMs. As in the example of the prediction of O-glycosylation site, presented in Supplementary Figure S7, the benchmark dataset concerning protein O-glycosylation, comprising 529 positive sites and 10 797 negative sites from 292 proteins, were used to test four tools—NetOGlyc, GPP, GlycoEP and OGTSite. The results of testing using the benchmark dataset with unbalanced positive and negative sites indicate that GPP provides balanced Sn and Sp, while the other three tools yield high Sp and low Sn. Supplementary Table S8 provides the testing results in detail. The non-homologous benchmark dataset can be utilized as an independent testing dataset in the prediction of PTM sites.

Table 2 lists advances and new features that are supported in dbPTM 2016. Future work is likely to support the growth of dbPTM as more data in research articles on MS/MS-identified modified peptides becomes available. To provide more information for disease analysis, the associations of diseases with PTM sites will be manually curated using an enhanced full-text mining system. Although this update supports a network analysis for a group of proteins, designing a uniform scheme that does so for all PTM types is difficult. Therefore, online resources for investigating the networks of a specific PTM type should be integrated into dbPTM. A future survey of how PTM sites affect the drug-binding affinity based on protein tertiary structures would significantly improve dbPTM.

**Table 2.** Advances and improvements in dbPTM 2016.

Features	dbPTM 1.0	dbPTM 3.0	dbPTM 2016
Publication	Nucleic Acids Res. 2006	Nucleic Acids Res. 2013	-
Protein entry	UniProtKB/Swiss-Prot (release 46)	UniProtKB release 2012-04	UniProtKB release 2015-05
Experimental PTM resource	UniProtKB/Swiss-Prot, Phospho.ELM and O-GLYCBASE	UniProtKB/Swiss-Prot, Phospho.ELM, PHOSIDA, HPRD, O-GLYCBASE, UbiProt, PhosphoSitePlus and PupDB	UniProtKB/Swiss-Prot, Phospho.ELM, PHOSIDA, HPRD, O-GLYCBASE, UbiProt, PhosphoSitePlus, PupDB, dbSNO, dbGSH and CPLM
Literature survey of PTMs	None	More than 3000 PTM peptides from approximately 250 articles	More than 12 000 modified peptides from approximately 500 articles
Computationally predicted PTMs	Phosphorylation, glycosylation and sulfation	20 types of PTM	20 types of PTM
Benchmark dataset	None	None	Yes
Integrative platform for PTM analyses	None	None	Integrating 71 databases and 116 tools associated with PTM analyses
Structural properties of PTM sites	Amino acid frequency	Amino acid frequency, solvent accessibility, secondary structure and intrinsic disorder region	Amino acid frequency, solvent accessibility, secondary structure, spatial amino acid composition, structurally neighboring amino aicds and side chain orientation
Protein–protein interaction	None	DIP (70), MINT (71), IntAct (72), HPRD and STRING (73)	Over ten PPI databases
Disease association of modified proteins	None	None	Yes
Drug association of PTM sites	None	None	Over 1100 PTM sites associated with drug binding
Network analysis	None	None	Cytoscape, KEGG metabolic pathway and protein–protein interactions
Graphical visualization	PTM, solvent accessibility, secondary structure, protein variation and protein domain	PTM, solvent accessibility, secondary structure, protein variation, protein domain, tertiary structure, orthologous conserved regions, sequence logo, substrate site specificity, substrate motifs and tertiary structure of PTMs	PTM, solvent accessibility, secondary structure, protein variation, protein domain, tertiary structure, orthologous conserved regions, sequence logo, substrate site specificity, substrate motifs, tertiary structure of PTMs, network analysis, spatial amino acid composition, structurally neighboring amino acids and side-chain orientation

## AVAILABILITY

The data content in dbPTM will be maintained and updated quarterly by continuously surveying the public resources and research articles. Also, the PTM data involved in diseases and drug-binding sites will be semiannually updated by database screening. The updated resource is now freely accessed online at http://dbPTM.mbc.nctu.edu.tw/. All of the experimentally verified PTM sites as well as the benchmark dataset can be downloaded in the text format.
